# Adjuvant interferon alpha 2b in high risk melanoma – the Scottish study

**DOI:** 10.1054/bjoc.2000.1623

**Published:** 2001-05

**Authors:** D A Cameron, M C Cornbleet, R M Mackie, J A A Hunter, M Gore, B Hancock, J F Smyth, on behalf of the Scottish Melanoma Group

**Affiliations:** Western General Hospital, Crewe Road South, Edinburgh; Western Infirmary, Dumbarton Road, Glasgow; Royal Infirmary, Lauriston Place, Edinburgh; Royal Marsden Hospital, Downs Road, Surron Surrey; Weston Park Hospital, Whitham Road, Sheffield, UK

**Keywords:** adjuvant, interferon, melanoma, Scottish trial

## Abstract

In 1989, the Scottish melanoma group initiated a randomized trial, comparing observation alone with 6 months' therapy with low dose interferon α (given subcutaneously 3 MU day^–1^, thrice weekly), for patients with primary melanomas of at least 3 mm Breslow thickness, or with evidence of regional node involvement. The trial was closed in 1993 with only 95 eligible patients randomized. There were no toxic deaths, and no patient failed to complete the treatment for reasons of toxicity. 6 months' treatment with low-dose interferon-α resulted in a statistically significant improved disease-free survival for up to 24 months after randomization (*P*< 0.05). However, at a median follow-up of over 6 years, although there was an apparent improvement in disease-free survival (from 9 to 22 months), and overall survival (from 27 to 39 months), consistent with larger studies powered to detect such differences, these differences were not statistically significant. The data therefore suggest that 6 months of low-dose interferon is active, and confirm the importance of the large randomized studies, such as the UKCCCR AIM-High and EORTC trials, that seek to confirm a possible survival advantage for low or intermediate dose interferon. © 2001 Cancer Research Campaign http://www.bjcancer.com


*British Journal of Cancer* (2001) **84**(9), 1146–1149
© 2001 Cancer Research Campaign
doi: 10.1054/ bjoc.2000.1623, available online at http://www.idealibrary.com on IDEAL®

http://www.bjcancer.com

### Adjuvant interferon alpha 2b in high risk melanoma – the Scottish study

**DA Cameron[1], MC Cornbleet[1], RM Mackie[2], JAA Hunter[3], M Gore[4], B Hancock[5] and JF Smyth[1], on behalf of the Scottish Melanoma Group**

[1]Western General Hospital, Crewe Road South, Edinburgh; [2]Western Infirmary, Dumbarton Road, Glasgow; [3]Royal Infirmary, Lauriston Place, Edinburgh; [4]Royal Marsden Hospital, Downs Road, Surron, Surrey; [5]Weston Park Hospital, Whitham Road, Sheffield, UK

**Summary** In 1989, the Scottish melanoma group initiated a randomized trial, comparing observation alone with 6 months' therapy with low dose interferon α (given subcutaneously 3 MU day$^{-1}$, thrice weekly), for patients with primary melanomas of at least 3 mm Breslow thickness, or with evidence of regional node involvement. The trial was closed in 1993 with only 95 eligible patients randomized. There were no toxic deaths, and no patient failed to complete the treatment for reasons of toxicity. 6 months' treatment with low-dose interferon-α resulted in a statistically significant improved disease-free survival for up to 24 months after randomization ($P < 0.05$). However, at a median follow-up of over 6 years, although there was an apparent improvement in disease-free survival (from 9 to 22 months), and overall survival (from 27 to 39 months), consistent with larger studies powered to detect such differences, these differences were not statistically significant. The data therefore suggest that 6 months of low-dose interferon is active, and confirm the importance of the large randomized studies, such as the UKCCCR AIM-High and EORTC trials, that seek to confirm a possible survival advantage for low or intermediate dose interferon. © 2001 Cancer Research Campaign http://www.bjcancer.com

**Keywords**: adjuvant; interferon; melanoma; Scottish trial

The incidence of malignant melanoma has increased faster than that of other solid tumours, and, despite successful surgery, patients with AJCC stage IIb (T4N0) or stage III (N1) melanoma remain at high risk of subsequent relapse and death (Mackie et al, 1992). Although many studies have investigated the possible benefits of adjuvant systemic therapy in this disease, there remains no universally accepted adjuvant regimen. The use of adjuvant interferon-α has been shown in at least three studies to improve the disease-free interval (Rusciani et al, 1997; Grob et al, 1998; Pehamberger et al, 1998), and possibly also overall survival (Kirkwood et al, 1996). However the optimum dose and schedule remain unclear, both in the adjuvant setting and in advanced disease, where response to systemic interferon is associated with therapy for at least 3 months, and in doses of at least $1 \times 10^6$ IU three times a week (Legha, 1986). Toxicity, however, does appear to be related to both dose and duration. As a result, the adjuvant trials conducted have not used the same regimen or dosage, but either high doses for up to one year, or at least a year's therapy with lower doses of interferon. Before any of these studies were reported, the Scottish Melanoma group conducted a prospective, randomized trial to test the possible disease-free and overall survival advantage of a short, 6-month course of low-dose interferon-α for patients with high-risk, surgically resected, malignant melanoma.

*Received 19 July 2000*
*Revised 8 November 2000*
*Accepted 21 November 2000*

*Correspondence to:* D Cameron

### PATIENTS AND METHODS

Patients with histologically proven malignant melanoma were eligible for randomization if considered at high risk of recurrent disease. This was defined as a primary lesion of at least 3 mm Breslow thickness (pT3b or pT4, AJCC Stage II), or following complete resection of regional lymph involvement detected clinically and confirmed pathologically (N1/N2, AJCC stage III). Patients were excluded if there was evidence of distant metastatic disease at presentation, a history of prior malignant disease (except basal cell carcinoma of the skin or surgically treated early carcinoma of the cervix), or if they had poor performance status or vital organ function. The local ethics committees of the participating hospitals approved the study.

After giving signed, informed consent, patients were randomized in a 1:1 ratio, with stratification by disease stage (AJCC stage II or III), to observation or treatment with alpha-Interferon 2b for 6 months. Interferon-α 2b, supplied by Schering-Plough, was given in a dose of $3 \times 10^6$ units subcutaneously 3 times a week for 6 months. Patients in both the observation and treatment arms were seen monthly for 6 months, with liver function tests and chest X-rays repeated every 3 months. Thereafter patients were followed up 3 monthly for up to 5 years, and at the institution's discretion thereafter. Any clinical suspicion of relapse was confirmed, as appropriate, by radiological, cytological or histopathological examination. Date of relapse was taken to be the date of first clinical suspicion of relapse if subsequently confirmed. Follow-up data on the surviving patients have been obtained from the institutions or the Scottish melanoma group database according to where the patient was last seen. Cause of death has been assumed to be due to melanoma if recurrence was either suspected or confirmed, or unless the treating institution confirmed an alternative proven cause.

The relapse rate was anticipated to be 60% in the control arm (assumed to be 40% for eligible stage II patients and 80% for stage III patients). Therefore, if this were to be reduced to 40% in the treatment arm with an 80% power, 120 patients were required to be followed to relapse. With these same assumptions therefore, the target recruitment was 240 patients. However accrual was much slower than anticipated, despite the inclusion of centres outwith Scotland, and the study was closed after almost 5 years with just under 100 patients enrolled.

### RESULTS

A total of 96 patients were enrolled in this study from 9 centres. 49 patients were randomized to observation alone and 47 to 6 months' treatment with interferon-α. 1 patient in the control arm has been lost to follow-up, and 1 patient randomized to interferon was found to be ineligible before any treatment was given. For the remaining 94 patients, the median follow-up is 6.5 years, with a maximum of 9.7 years.

### Disease-free and overall survival

Figures 1 and 2 show that both the disease-free and overall survival appear to be consistently better for the patients treated with interferon-α as compared with the observation only group. The median disease-free survival for interferon-treated patients was 22 months, as compared with 9 months for the control group, with a concomitant improvement in overall survival from 27 to 39 months for the interferon-α treated patients. Furthermore this same trend was to be seen for patients with both stage II and stage III disease (data not shown). However these apparent differences, although if real of clinical significance, are not statistically significant. There was in addition no significant difference in the site of first relapse between the two groups. When analysing the disease-free survival data at early time points, statistically significant differences in favour of interferon are to seen at 6, 12, 18 and 24 months ($P < 0.05$, $P < 0.025$, $P < 0.025$ and $P = 0.05$, respectively). Figure 3 therefore shows the hazard rate for disease-free survival at 6-month intervals for the whole duration of available follow-up, confirming a clear early benefit for adjuvant interferon-α extending for up to 24 months from trial entry. When considering the patients with stage II and stage III disease separately, this early difference in hazard rate for interferon-α treated patients is also seen (data not shown). Furthermore, Figure 3 suggests that there might be a further benefit for adjuvant interferon-α in the form of reduced hazard rate for late relapse, although this observation is based on data from only 6 patients.

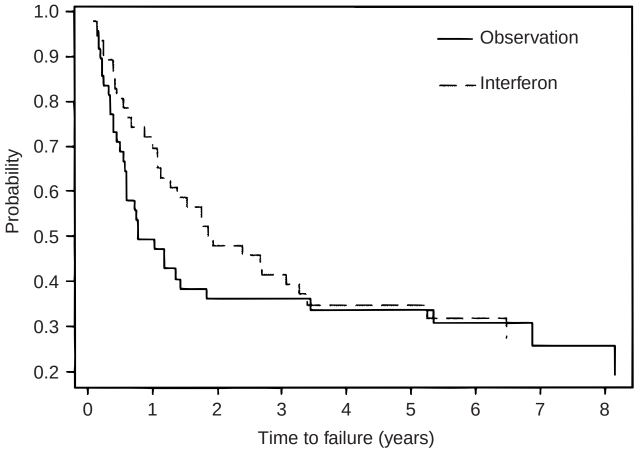

**Figure 1** Disease-free survival by treatment arm (Observation $n = 48$, Interferon $n = 46$). $\chi^2 = 1.9$, $P > 0.1$

### Toxicity

Of the 46 eligible patients randomized to receive adjuvant interferon, 29 (63%) completed the 6 months as per protocol. 3 (7%) experienced a dose reduction, 2 for neutropenia and 1 for drug-related fever. Treatment was stopped in 13 (28%) following confirmed recurrence, including one who had experienced a dose reduction. 2 patients (4%) randomized to the treatment arm did not receive treatment, but have been included in the analyses on an intent-to-treat basis. The treatment was in general well tolerated, with patients able to continue in full-time work. Paracetamol was given as prophylaxis for the most frequently reported side effects of myalgia and chills, and was in general discontinued early in the course of therapy.

### DISCUSSION

This study demonstrates that adjuvant interferon-α, given at low dose for only 6 months, appears to have some impact on high-risk

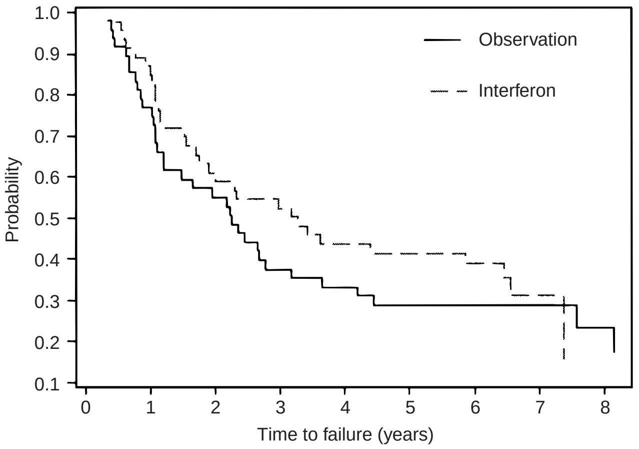

**Figure 2** Overall survival by treatment arm (Observation $n = 48$, Interferon $n = 46$). $\chi^2 = 1.5$, $P > 0.2$

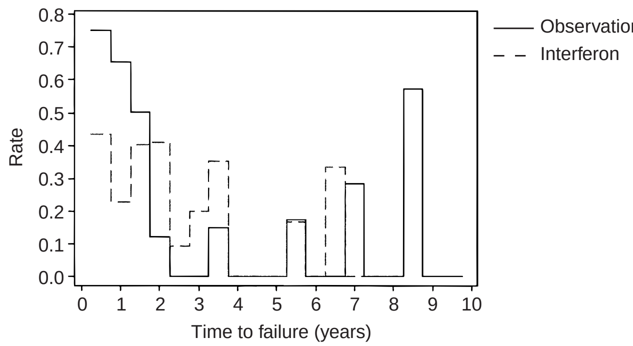

**Figure 3** Hazard ratio for relapse by time, with an interval of 6 months

*© 2001 Cancer Research Campaign*

stage II and III melanoma. There was an improvement in disease-free survival maintained for more than a year beyond the cessation of a well-tolerated adjuvant therapy. The lack of any statistically significant benefit on long-term disease-free and overall survival could in part be a result of the disappointing recruitment, resulting in a trial underpowered to detect the hypothesized treatment effect. If the planned number of patients had been accrued, and the same improvement in disease-free survival seen, then statistical significance could have been achieved. Therefore, before concluding that the study is *clinically* negative, the data need to be considered in the context of previously reported, larger, studies.

The first data to suggest that adjuvant interferon could impact on the natural history of malignant melanoma came from the WHO adjuvant study. In this trial, 426 evaluable patients with involved lymph nodes were randomized to either an observation group or 3 years' treatment with Interferon-α 2a at the dose of 3 MU day$^{-1}$ thrice weekly (Cascinelli, 1995). However more recent analyses, with longer follow-up, have not confirmed the disease-free survival benefit that had been reported earlier (Cascinelli, 1999). In contrast, the data from the ECOG E1684 study demonstrate a clear disease-free and an apparent survival advantage for the use of high-dose interferon-α in high-risk melanoma, using a one-sided *P* value, the justification for which is not given in the text (Kirkwood et al, 1996). This study randomized 287 patients with stage II and III disease between observation only, and an experimental arm comprising 4 weeks of 20 MU interferon-α given intravenously 5 days week$^{-1}$, followed by 48 weeks of 10 MU interferon-α given subcutaneously 3 times a week. The majority of patients were unable to complete the schedule, and there were 2 toxic deaths on the treatment arm. There was a clear disease-free survival advantage for interferon-α, but using the gold standard of an intention-to-treat analysis and a *two-sided test*, the difference in overall survival has a *P* value between 0.05 and 0.06. Furthermore, 89% of patients had stage III disease, and thus no conclusions can be drawn from this study about the management of high-risk node-negative melanoma. In contrast, 2 studies have reported a disease-free survival advantage for prolonged low-dose interferon. A French study randomized 489 eligible patients with stage II disease between observation and 18-months of 3 MU interferon-α given subcutaneously 3 times a week (Grob et al 1998). There was a significant disease-free survival advantage for the patients who received interferon-α, and a trend for an improvement in overall survival with a two-sided *P* value between 0.05 and 0.06. The Austrian study randomized 311 patients with stage II disease between observation and one year's treatment with 3 MU interferon-α (Pehamberger et al, 1998). The authors reported a clear disease-free survival advantage for the patients given interferon, but there were insufficient deaths observed during the relatively short follow-up period to evaluate any effect on survival. The next ECOG study, E1690, compared the high-dose Kirkwood regimen with 2 years of 3 MU interferon given subcutaneously 3 times a week, and an observation only group (Kirkwood et al, 2000a). In contrast to the earlier E1684 study, E1690 has not demonstrated a survival advantage for either treatment arm as compared to the control observation arm, but has reported a statistically significant disease-free survival advantage for the high-dose regimen as compared with the observation arm (hazard ratio 1.28, $P = 0.05$). Furthermore, an early analysis of data from the subsequent ECOG trial 1694, which compares 2 years' high-dose interferon with a ganglioside GM2 vaccine, again suggests a survival advantage for interferon over the alternative therapy (hazard ratio 1.52, $P < 0.01$) (Kirkwood et al, 2000b). Finally, it must be noted that the ECOG studies employed interferon-α-2b, the compound used in this study, whereas in the French and Austrian studies, patients were treated with the alternative form, interferon-α-2a. Whether there is a significant difference in effect between these two forms of interferon-α remains however to be proven.

The studies that have reported since the Scottish study was closed to recruitment consistently demonstrate a clear improvement in disease-free survival for treatment with interferon-α, and furthermore 2 studies, one each with low-and high-dose interferon-α, report an improvement in overall survival with a two-sided *P* value between 0.05 and 0.06 (Kirkwood et al, 1996; Grob et al, 1998). Furthermore, the magnitudes of these improvements are well within the confidence intervals for the observed disease-free and overall survival rates in the current study. The pattern over time seen for the hazard-rate for relapse with 6 months low-dose interferon-α in the Scottish study (see Figure 3), is similar to that reported by Grob for 18 months of the same treatment, with a significant early effect from therapy. However the current study reports longer-term follow-up data, and there is the suggestion in both stage II and stage III disease for a late benefit. The only other study to report similar long follow-up is the E1684, and although there is no such trend overall, a possible additional late difference in hazard rate is seen in some sub-groups, including the small number of patients with stage II disease. The biological rationale for these observations would be that there is an immediate reduction in relapse consequent upon the cytotoxicity of interferon-α, whereas one might anticipate that any immunomodulant effect on microscopic disease would be better seen in the pattern of late relapse.

Thus the data in the Scottish adjuvant melanoma trial are consistent both with the results of the other adjuvant studies that have been fully reported to date, and with the notion that adjuvant interferon-α gives at least a disease-free survival advantage for patients with both stage II and stage III disease. The optimal duration of therapy remains unclear, and it cannot be concluded with certainty that either low- or high-dose interferon-α gives a definite survival advantage, so there remains no standard adjuvant therapy. The results of the larger trials randomizing patients between low or intermediate dose interferon-α and observation, such as AIM-High and the EORTC trials, will be better powered than the prior studies to answer this all-important question of a possible survival advantage for adjuvant interferon in the treatment of high-risk surgically resected malignant melanoma.

### ACKNOWLEDGEMENTS

This study could not have been conducted without the hard work of the data managers Caroline MacDonald and Gillian Smith, and the other clinicians recruiting patients into the study: Dr Graham Brown, Leicester Royal Infirmary; Dr Norris, Dumfries and Galloway Royal Infirmary; Prof P Selby, Leeds. Finally, of course, the most important contribution was made by the patients willing to be randomized within the trial.

### REFERENCES

Cascinelli N (1995) Evaluation of efficacy of adjuvant rlFN alpha 2a in melanoma patients with regional node metastases (Meeting abstract). *Proc Annu Meet Am Soc Clin Oncol* **14**: A1296

© 2001 Cancer Research Campaign

Cascinelli N (1999) Data presented at 3rd International Conference on Adjuvant Therapy of Malignant Melanoma. London.

Grob JJ, Dreno B, de la Salmoniere P, Delaunay M, Cupissol D, Guillot B, Souteyrand P, Sassolas B, Cesarini JP, Lionnet SL, Chastang C and Bonerandi JJ (1998) Randomised trial of interferon alpha-2a as adjuvant therapy in resected primary melanoma thicker than 1.5 mm without clinically detectable node metastases. French Co-operative Group on Melanoma. [see comments]. *Lancet* **351**: 1905–1910

Kirkwood JM, Strawderman MH, Ernstoff MS, Smith TJ, Borden EC and Blum RH (1996) Interferon Alfa-2b adjuvant therapy of high-risk resected cutaneous melanoma: the Eastern Co-operative Oncology Group Trial EST 1684. *J Clin Oncol* **14**: 7–17

Kirkwood JM, Ibrahim JG, Sondak VK, Richards J, Flaherty LE, Ernstoff MS, Smith TJ, Rao U, Steele M and Blum RH (2000a) High- and low-dose interferon alfa-2b in high-risk melanoma: first analysis of intergroup trial E1690/S9111/C9190. *J Clin Oncol* **18**: 2444–2458

Kirkwood JM, Ibrahim J, Sondak VK, Sosman JA and Ernstoff MS (2000b) Relapse-free and overall survival are significantly prolonged by high-dose IFN alpha 2b (HDI) compared to vaccine GM2-KLH with QS21 (GMK, Progenics) for high-risk resected stage IIB-III melanoma: Results of the Intergroup Phase III study E1694/S9512/C503801. *Eur J Cancer* 11 (Supp 4): abstract 40.

Legha SS (1986) Interferons in the treatment of malignant melanoma. A review of recent trials. *Cancer* **57**: 1675–1677

Mackie R, Hunter JA, Aitchison TC, Hole D, Mclaren K, Rankin R, Blessing K, Evans AT, Hutcheon AW and Jones DH (1992) Cutaneous malignant melanoma, Scotland, 1979–89. The Scottish Melanoma Group. *Lancet* **339**: 971–975

Pehamberger H, Soyer HP, Steiner A, Kofler R, Binder M, Mischer P, Pachinger W, Auböck J, Fritsch P, Kerl H and Wolff K (1998) Adjuvant interferon Alfa-2a treatment in resected primary stage II cutaneous melanoma. *J Clin Oncol* **16**: 1425–1429

Rusciani L, Petraglia S, Alotto M, Calvieri S and Vezzoni G (1997) Postsurgical adjuvant therapy for melanoma. Evaluation of a 3-year randomized trial with recombinant interferon-alpha after 3 and 5 years of follow-up. *Cancer* **79**: 2354–2360

*© 2001 Cancer Research Campaign*
